# Clinical Methods Supporting Initial Recognition of Early Post-Stroke Seizures: A Systematic Scoping Review

**DOI:** 10.3390/neurolint17100159

**Published:** 2025-10-03

**Authors:** Clare Gordon, Hedley C. A. Emsley, Catherine Elizabeth Lightbody, Andrew Clegg, Catherine Harris, Joanna Harrison, Jasmine Wall, Catherine E. Davidson, Caroline L. Watkins

**Affiliations:** 1 School of Nursing and Midwifery, University of Lancashire, Preston PR1 2HE, UK; celightbody@uclan.ac.uk (C.E.L.);; 2 Lancashire Teaching Hospitals NHS Foundation Trust, Preston PR2 9HT, UK; hedley.emsley@lancaster.ac.uk; 3 Lancaster Medical School, Lancaster University, Lancaster LA1 4YW, UK; jasmine.wall2@srft.nhs.uk; 4 Health Technology Assessment Unit, University of Lancashire, Preston PR1 2HE, UK; aclegg3@lancashire.ac.uk (A.C.); charris10@lancashire.ac.uk (C.H.); jharrison12@lancashire.ac.uk (J.H.)

**Keywords:** stroke, epileptic seizures, electroencephalogram, clinical recognition, scoping review, stroke units

## Abstract

**Background:** Stroke is a leading cause of seizures and epilepsy, both of which are linked to increased mortality, disability, and hospital readmissions. Early recognition and management of seizures in acute stroke are crucial for improving outcomes. Electroencephalogram (EEG) is not routinely used for post-stroke seizure monitoring and is typically initiated only after clinical suspicion arises, making bedside recognition essential. This scoping review aimed to map the existing literature on clinical methods used for identifying and observing early post-stroke seizures (EPSSs) at the bedside. **Methods:** We included literature involving adults with acute ischaemic stroke or primary intracerebral haemorrhage who were diagnosed or suspected of having inpatient EPSS. Searches were conducted in Medline, CINAHL, Embase, and the Cochrane Library for English-language publications up to April 2023. Eligible sources included primary research, case reports, systematic reviews, clinical guidelines, consensus statements, and expert opinion. Reference lists of included articles were also reviewed. Data were charted and synthesised to assess the scope, type, and gaps in the evidence. **Results:** Thirty papers met inclusion criteria: 17 research studies, six expert opinions, four case reports, and three clinical guidelines. Empirical evidence on clinical methods for seizure recognition and monitoring in acute stroke was limited. No studies evaluated the effectiveness of different approaches, and existing recommendations lacked detail and consensus. **Conclusions:** Accurate EPSS diagnosis is vital due to its impact on outcomes. This review highlights inconsistency in monitoring methods and a clear need for targeted research into effective clinical identification strategies in acute stroke care.

## 1. Introduction

Early post-stroke seizures (EPSSs), also termed provoked or acute symptomatic seizures, describe seizures provoked by acute brain injury due to stroke. These are different to post-stroke epilepsy that arises from long-term brain changes after stroke and seizures which are unprovoked by any other factor, such as metabolic, toxic etc. [1,2]. There is a lack of consensus in the literature on the definition and timing of what is classed as EPSS. The International League Against Epilepsy (ILAE) defines acute symptomatic seizures as occurring up to 7 days after acute stroke onset [3], whereas, in the literature, EPSS is defined as occurring between 48 h and 2 weeks after stroke [1,4].

Post-stroke seizures, occurring in the immediate aftermath of an acute stroke, can complicate diagnosis and management, and can even go unrecognised by clinicians and patients [5,6]. These seizures can present as new or unexplained reduced consciousness, persistent focal neurological deficits (e.g., due to postictal paresis), or exacerbation of existing deficits, potentially leading to inaccurate assessment of stroke severity or the attribution of symptoms to stroke recurrence. Timely recognition and treatment of seizures are essential to prevent further injury to already compromised brain tissue and to avoid the inappropriate use of stroke-specific therapies when symptoms are misattributed [7]. It is important to identify and diagnose EPSS as some studies have demonstrated associations with increased mortality, disability and recurrent hospital admissions [8,9]. Moreover, the risk of developing post-stroke epilepsy is significantly higher in patients who experience EPSS compared to those who do not [10]. Accurate detection of EPSS may also facilitate early discussions with patients regarding their long-term seizure risk.

EPSSs are more likely to occur after intracerebral haemorrhage (prevalence 10–16% across stroke populations) but are also common after ischaemic stroke (prevalence 3–15%) [1,11]. The risk of EPSS is increased in ischaemic stroke with haemorrhagic transformation, with cortical involvement and with increasing stroke severity [2,12]. Numerically, given the epidemiology of pathological stroke subtypes, early post-ischaemic stroke seizures will be more frequently encountered in the acute stroke context.

Electroencephalogram (EEG) is a helpful tool in detecting seizure activity, recognising non-convulsive status epilepticus and predicting seizure recurrence [13]. However, EEG is not 100% sensitive, nor used systematically for post-stroke seizure monitoring due to time and resource limitations, and it is usually instigated after seizures are suspected [14]. Typically, UK centres have access to up to 30 min video EEG recording, which can fail to capture seizures if they are not occurring during the recording, although a propensity towards seizure can sometimes be observed [13,15]. Therefore, interpretation of events, with or without EEG, is highly dependent upon clinical acumen and identification of clinical signs. With the lack of reference specifically on recognition and monitoring for EPSS in national guidance, this study aimed to map the breadth of evidence in relation to clinical methods used to support the identification and monitoring of EPSS. Our research question was, what is known from the existing literature about the clinical methods used for identifying and monitoring seizures in adults being treated for acute stroke?

## 2. Materials and Methods

The review was guided by Arksey and O’Malley’s framework, which includes the following stages: (i) identifying the research question; (ii) searching for relevant papers; (iii) selecting papers; (iv) charting the data; (v) collating, summarising, and reporting the results [16,17]. The review protocol has been registered with OSF and published online (OSF ID: bkejc) [18].

### 2.1. Identifying the Research Question and Eligibility Criteria

The research question was developed with input from subject experts comprising academics, clinicians, and patient advisors for the identification of relevant outcomes. We included literature published up to April 2023, encompassing all study types, including systematic reviews with meta-analysis and non-research literature such as clinical guidelines and consensus statements. We included literature on adults (≥18 years) with acute stroke (ischaemic or primary intracerebral haemorrhage) and seizures that occurred in hospital, within 2 weeks of stroke onset. We included seizures occurring at stroke onset and seizures occurring with an acute stroke intervention such as reperfusion therapies. We excluded literature reporting on seizure as a stroke mimic, patients with known epilepsy or seizures before their stroke, and patients with diagnoses of subdural and subarachnoid haemorrhages or cerebral ischaemia without arterial circulation obstruction, such as vasospasm or secondary to trauma. We excluded papers published in languages other than English. See Table 1 for the PICo criteria [19] for the review question.

### 2.2. Searching for Relevant Papers

MEDLINE (Ovid), CINAHL (EBSCOhost), EMBASE (Ovid), and the Cochrane Library (all databases via Wiley) were originally searched in 2021 and updated on 21 April 2023. To ensure that all relevant information was captured, we also searched a variety of grey literature sources (searched January 2023)—Grey Literature Report, OpenGrey, and Web of Science Conference Proceedings—to identify studies, case reports, and conference abstracts of relevance to this review. We also conducted a targeted search, using Google, of the grey literature, and we specifically searched national and international organisations’ websites with an interest in stroke and/or seizures, such as the Stroke Association, the Epilepsy Society, the ILAE, the British and Irish Association of Stroke Physicians, the European Stroke Organisation, and the American Stroke Association. A hand search was conducted using the reference lists of included papers to identify additional relevant papers. The search strategy was developed and piloted by an information specialist (CH) with input from the project team. The search strategies are provided in the Supplementary Materials (Supplementary Material 1) and are published online [18].

### 2.3. Selection of Sources of Evidence and Charting the Data

Following the searches, duplicate records were removed in EndNote before results were uploaded into Rayyan© online collaborative systematic review software [20] for record management and title and abstract screening. A two-part screening process against the inclusion criteria was used: (a) a title and abstract review, and (b) a full text review.

**Figure 1:**
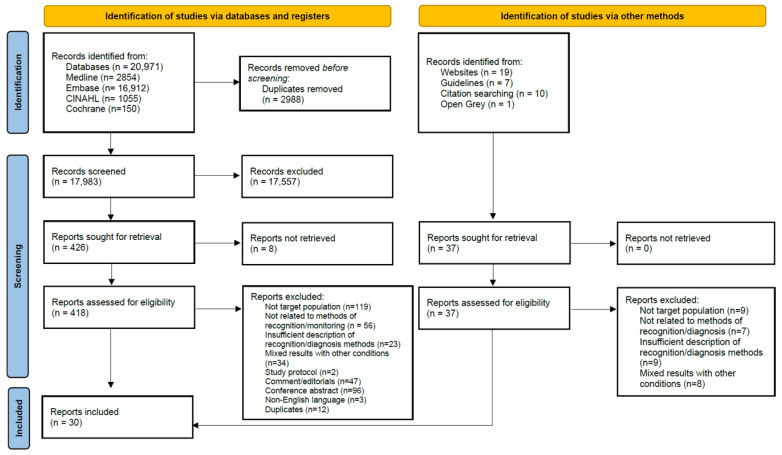
PRISMA flow diagram.

Title and abstract screening were conducted mainly by one reviewer, with 1000 citations independently screened by two reviewers (C.G. & J.W.) with 91.5% agreement between reviewers. Full text papers were assessed against the inclusion criteria; reasons for exclusion were recorded and are reported in the results by one reviewer. Any disagreements that arose were resolved through discussion with the wider project team. The results of the search selection are reported using the Preferred Reporting Items for Systematic Reviews and Meta-Analyses extension for Scoping Reviews (PRISMA-ScR) (flow diagram in Figure 1) [21]. The completed PRISMA-ScR checklist is available in the Supplementary Material (Table S1).

### 2.4. Data Charting Process

We developed and piloted our data charting form with evidence synthesis experts (J.H. & A.C.) based on scoping review methodology [16]. The piloting process included data charting of three papers independently, with comparison of accuracy and comprehension after completion. The charting of the data was divided between the three reviewers (C.G., J.W., C.E.D.). Where results of the same study were reported in more than one publication, we collated the results and used the publication with the most data relevant to our research question as the primary reference. Data charted included type of paper (e.g., primary research, conference proceedings, clinical guideline), study aims, methods, clinical assessment method, participants, study location, study setting, type of stroke, type of seizure, and key results relevant to our research question (e.g., sensitivity and specificity of tool). Due to inconsistencies in EEG terminology across studies, we systematically charted the terminology used by authors, along with reported technical parameters and recording durations. Where there was no reference to video data in the methods or results, we assumed video was not incorporated. Finally, a scoping review does not typically involve a quality assessment and therefore we did not appraise the quality of evidence [17].

## 3. Results

### 3.1. Selection of Sources of Evidence

We included 30 papers, 26 from database searches and four retrieved from alternative sources and citation searching. The selection process is outlined in the PRISMA diagram (Figure 1). Dates of publication ranged from 1996 to 2023. The definition of EPSS ranged from 24 h to 1 month after stroke onset. Papers originated from 13 different countries, 18 (60%) from Europe (Belgium (2), France (1), Germany (1), Italy (4), Poland (1), Portugal (2), Sweden (2), Switzerland (2), Turkey (3)), nine (30%) from the United States of America (USA), two (7%) from Asia (China (1), South Korea (1)) and one (3%) from Africa. A summary of the key characteristics are in Table 2.

For reporting the results, we tabulated the papers according to the traditional hierarchy of evidence [22] (see Table 3, Table 4 and Table 5). Table 3 summarises the characteristics of 10 included papers consisting of expert opinions and case reports that are considered low-quality evidence in the hierarchy of evidence [22]. Apart from Green et al.’s clinical guidelines [23] (Table 5), these 10 papers provided the most detail on the nursing contribution to seizure observation and supporting bedside EEG monitoring in critical care settings.

Seventeen primary research papers were identified (Table 4). All were observational design and eight used retrospective data collection [31,33,34,35,36,37,38,39]. Most patients were recruited from centres providing stroke specialist services. Three studies recruited patients from intensive care [31,39,40]. Two studies did not describe the type of specialist stroke service provision [36,41]. Sample sizes ranged from 48 to 3344. Eleven papers (64.7%) had <500 participants, four papers (23.5%) had 500–1000 participants, and two papers (11.8%) had >1000 participants (Table 2). Ethnicity was not consistently reported. Of the participants recruited, 83.7% (*n* = 8111) had a diagnosis of ischaemic stroke and 54.7% (*n* = 5302) were male.

**Table 4:** Primary research studies detailing methods used for the identification and observation of early post-stroke seizures.

First Author, Year	Country	Study Type	Study Aim	Number of Participants	Male (%)	Ischaemic Stroke (%)	Clinical Method to Identify Seizure
**Ba, 2021** [33]	France	Cohort	To evaluate whether thrombolysis is associated with an increased risk of early epileptic seizures in a cohort of consecutive patients who underwent an angiography in emergency care for cerebral ischaemia due to large-vessel occlusion	1638	783 (48%)	1638 (100%)	Clinical diagnosis. EEG used to diagnose seizures with atypical manifestation. EEG not used systematically.
**Beghi, 2011** [42]	Italy	Cohort	To identify incidence and predictors of acute symptomatic seizures in a cohort of patients with first stroke	714	399 (56%)	609 (85%)	Direct observation by medical staff or reliable witness. Simple loss of consciousness or short episodes of confusion excluded. EEG only when indicated by medical staff.
**Belcastro, 2014** [43]	Italy	Cohort	To evaluate in a stroke unit the usefulness of a prolonged, at least 6 h, video-EEG recording (VEEG) in identifying episodes of non-convulsive status epilepticus after an acute ischemic stroke	889	566 (64%)	889 (100%)	Prolonged VEEG routinely within first 7 days of admission or immediately upon suspected seizure activity
**Bentes, 2017** [44]	Portugal	Cohort	To compare the frequency of seizures and EEG abnormalities between stroke patients treated and not treated with thrombolysis	151	89 (59%)	151 (100%)	Continuous VEEG in first 72 h, daily for first 7 days + if neurological worsening, at discharge
**Bentes, 2018** [13]	Portugal	Cohort	To investigate whether early EEG abnormalities are independent predictors of post-stroke epilepsy	151	112 (74%)	151 (100%)	Continuous VEEG in first 72 h, daily for first 7 days + if neurological worsening, at discharge
**Carrera, 2006** [45]	Switzerland	Case control	To determine the incidence and risk factors of electrical seizures and other electrical epileptic activity using cEEG in patients with acute stroke	100	58 (58%)	91 (91%)	cEEG routinely on first full admission day
**Daniele, 1996** [34]	Italy	Cohort	To evaluate the incidence of seizures and relationship between the various types of seizures and lesion location	217	125 (58%)	187 (86%)	Observation and description by either experienced departmental staff or by witness relatives of the patient
**Jung, 2012** [35]	Switzerland	Cohort	To analyse the influence of early and late epileptic seizures on the outcomes of patients with acute ischemic stroke treated with thrombolytic therapy	805	438 (56%)	805 (100%)	Symptom information from the patient, from a witness, or both
**Kim, 2016** [36]	South Korea	Cohort	To define clinical predictors of seizure recurrence after first post-stroke seizure in ischaemic stroke	48	29 (60%)	48 (100%)	Seizure diagnosed clinically. Standard EEG within 24–72 h of seizure onset.
**Lasek-Bal, 2023** [46]	Poland	Cohort	To determine the prevalence and nature of changes in EEG recordings from the stroke hemisphere and contralateral hemisphere	131	62 (47%)	131 (100%)	Two EEGs in first 72 h and one before discharge
**Mecarelli, 2011** [47]	Italy	Cohort	To analyse EEG patterns performed within 24 h of stroke onset	232	107 (46%)	177 (76%)	EEG within 24 h of admission
**Onder, 2017** [40]	Turkey	Cohort	To identify whether EEG findings could be a marker for post-stroke seizure development and survival in patients with acute ischemic or haemorrhagic stroke, who were followed up in a neurological intensive care unit	50	23 (46%)	37 (74%)	Continuous EEG in neurological intensive care unit
**Sarfo, 2020** [41]	Africa	Cohort	To assess the frequency and factors associated with post-stroke seizures by stroke types across 15 hospitals in Nigeria and Ghana	3344	1870 (66%)	2091 (62%)	Seizure diagnosed clinically. No EEG.
**Scoppettulo, 2019** [37]	Belgium	Cohort	To assess if epileptic activities were associated with neurological deterioration in acute ischaemic stroke	81	46 (56%)	81 (100%)	EEG
**Tako, 2022** [38]	Germany	Cohort	To analyse predictive factors for acute symptomatic seizures in a well-defined patient population who experienced an ischemic stroke due to large vessel occlusion and treated after mechanical recanalisation	979	509 (52%)	979(100%)	Clinically observed ictal stigmas. EEG only when indicated by medical staff.
**Vespa, 2003** [48]	USA	Cohort	To determine whether early seizures that occur frequently after intracerebral haemorrhage led to increased brain oedema	109	60 (55%)	46 (42%)	EEG within 24 h of stroke onset and admission to intensive care
**Yerram, 2019** [39]	USA	Cohort	To evaluate risk factors from examination, imaging, and cEEG for the development of seizures in critically ill patients with ICH	57	26 (46%)	0 (0%)	Continuous EEG at the order of the physician

**Table 5:** Clinical guidelines/recommendations for the identification and observation of early post-stroke seizures.

First Author, Year	Country	Clinical Setting	Clinical Method to Identify Seizure	Duration of Method	Indications
**Green, 2021** [23]	USA	Acute ischaemic stroke	Standardised approach to recognition, assessment, and documentation of the seizure Neurological examination EEG	Not reported	Monitor with EEG for change in mental status or depressed level of consciousness out of proportion to the stroke
**Hemphill, 2015** [49]	USA	Acute intracerebral haemorrhage	cEEG	At least 24 h	Depressed mental status out of proportion to the stroke
**Tatum, 2022** [50]	USA	Inpatient	Continuous VEEG monitoring	Condition-specific	Continuous VEEG should be used to differentiate between epileptic and non-epileptic events

Three clinical guidelines were identified (Table 5). Two papers were American Heart Association scientific statements [23,40]. Green et al. [23] provided guidance on observation for seizures within their nursing care scientific statement. Both Green et al. [23] and Hemphill et al. [40] outlined indications for EEG monitoring in acute stroke. A third guideline, jointly published by the International League Against Epilepsy and the International Federation of Clinical Neurophysiology, provided guidance on inpatient long-term VEEG monitoring for differentiation between epileptic and non-epileptic events. It did not provide any guidance specifically for stroke [50].

### 3.2. Clinical Methods for the Identification and Observation of Seizures

Our aim for this review was to map the available literature on methods used in the identification and observation of EPSS in inpatient settings. We identified one research paper evaluating the usefulness of EEG monitoring on a stroke unit [43]. No papers were identified that evaluated the accuracy of different clinical methods for identifying EPSS. Five types of clinical methods used in the identification and observation of EPSS were reported in the literature: (i) cEEG, (ii) periodic EEG, (iii) VEEG, (iv) clinical observation, and (v) family witness. There were inconsistencies in the terminology and definitions for EEG type and therefore we have used the same terminology used by the authors. cEEG (reported with or without concurrent video recording) received the most attention in the literature, including eight research studies evaluating EEG changes in EPSS [13,37,40,43,44,45,48] (Table 6). Indications for performing EEG also varied and are summarised in Table 7. Routine EEG at prescribed timepoints was mostly implemented in research papers. Case studies, expert opinion, and clinical guidance literature relied on clinical changes in neurological status to justify EEG investigation (Table 7). Only two papers reported using methods of seizure activation such as hyperventilation [44,46].

Table 5 provides a summary of the key information for each clinical method.

(i) cEEG. Seventeen papers, including eight research studies, referred to cEEG for EPSS detection and monitoring. Technical parameters for EEG were reported in nine papers with varying detail and no standardisation. cEEG was typically initiated at the earliest opportunity after suspected seizure or stroke onset. Duration of monitoring ranged from >6 h to 7 days; one intensive care study monitored for up to 38 days. Three papers described bedside cEEG visible to nursing staff [25,31,45], and two highlighted the need for nursing and physician competency in recognising electrographic seizure patterns [25,31]. Bautista [25] specified essential bedside EEG interpretation skills, including waveform frequency, amplitude, morphology, and symmetry. Two papers reported retrospective cEEG review by either trained physicians or electroencephalographers [31,45]. Several papers highlighted the utility of cEEG in detecting non-convulsive seizures and periodic discharges (associated with increased seizure risk) [2,28,31,32].
(ii) Periodic EEG. Eleven papers referred to periodic EEG, reported as 20 to 30 min in duration (Table 6). One paper [28] initiated an emergency EEG performed soon after stroke presentation due to fluctuant confusion, followed by cEEG. Two research papers used periodic EEG systematically on all acute stroke patients [46,47]. They were also the only papers reporting on technical parameters, both using the International 10–20 system with 14 [47] or 21 [46] electrodes. In one study, serial EEGs over several days were indicated if the first EEG showed abnormal epileptiform activity [47].
(iii) VEEG. Concurrent video recording with EEG is considered best practice, supplementing clinical assessments and linking electrographic seizures with clinical changes [2]. Tatum et al. [50] recommend a single camera setup and provide guidance on EEG and video synchronisation and digital memory requirements. Video use was reported in one out 11 papers using periodic EEG and in 10 out of 19 cEEG papers (Table 6). Mader’s case report [30] described a 28 s clonic seizure observed on video but obscured on EEG due to movement artefact. This case drew attention to the narrow time window for observing seizures if relying on human observation as well as the value of concurrent video recording with EEG.
(iv) Clinician observation. Nine papers, six research papers, addressed clinical observation in EPSS. In five papers, seizures were diagnosed clinically without details on observation procedures or staff training [34,35,38,41,42]. Among papers reporting criteria, seizure definitions and thresholds were varied [24,35,36]. The International League Against Epilepsy definitions were used in three papers [36,38,41]. Green et al. [23] recommended a standardised nursing observation approach for post-stroke complications, including seizures, but did not specify a method. Bautista [50] recommended a systematic assessment covering onset and duration, level of consciousness, eye deviation, gaze, pupil size, urinary incontinence, body movements, and motor function, with periodic assessments until baseline recovery [50].
(v) Family witness. Four research papers [34,35,41,42] and case report [30] referred to a family witness description contributing to the diagnosis of seizure at stroke onset or on hospitalisation. No papers referred to supplemental video information recorded on smart phones provided as part of the witness account.

## 4. Discussion

This scoping review has identified clinical and electrophysiological methods used in the identification and monitoring of EPSS. While we found no studies evaluating the effectiveness of these methods, we included papers that described seizure identification and monitoring methods within their research designs or practice recommendations. Many studies on seizure prevalence and characteristics in acute stroke were excluded because they lacked detail on how seizures were identified. Our review highlights a notable gap in the literature, particularly in nursing research, regarding the most effective and accurate methods for seizure monitoring in acute stroke patients.

This scoping review was undertaken to highlight knowledge gaps and areas requiring further research in relation to diagnostic approaches, in light of the lack of consensus on key definitions of EPSS in the literature. We aimed to include a wide range of literature, using a systematic search process in extensive databases and within grey literature, but it is possible that we have missed some relevant literature. We did not undertake a formal quality assessment, but we did chart data on methodological information that informed our interpretation of the evidence. We did exclude papers that had mixed early- and late-seizure onset participants or where onset of seizure after stroke was not clear.

We found significant heterogeneity in seizure identification and monitoring practices across research, clinical settings, and guidelines. Although consensus classifications such as the ILAE exist, they are not fully utilised in acute stroke care. This leads to variability in how clinical signs are interpreted and managed. We found EEG monitoring also lacked standardisation—indications for its use, technical parameters, duration and interpretation varied widely. This inconsistency hampers accurate estimation of EPSS prevalence and affects diagnostic accuracy, and, ultimately, patient outcomes.

A further layer of complexity is distinguishing seizure activity from the acute stroke itself, especially in the first hours post-stroke when seizure signs may be misattributed to stroke progression [39]. Several studies assumed that post-stroke seizures are readily recognised by clinicians, triggering EEG investigation. However, other papers challenge this assumption as short-duration focal seizures are more likely to go unnoticed by staff. Systematic EEG monitoring can reveal electrographic seizures with no clinical manifestations, non-convulsive status epilepticus, and specific patterns indicating heightened seizure risk [39,43,46,47]. This review identified justification for EEG in acute stroke, and further prospective research is needed into patient selection, type, and duration of EEG monitoring and its impact on treatment.

## 5. Conclusions

Research on the prevalence, diagnosis, and management of EPSS relies on effective recognition and observation of post-stroke patients for seizures. This scoping review highlights a significant gap in the literature on validated methods for identification and observation of seizures in acute stroke care. The absent of consistent methods may contribute to the underestimation of its prevalence, delayed diagnosis and treatment, and, ultimately, poorer outcomes. Greater attention to EPSS in both research and clinical practice is warranted. Our findings highlight a need for further clinical research to determine which methods, or combination of methods, can improve recognition rates of suspected seizure activity and ultimately improve diagnostic accuracy.

## Figures and Tables

**Table 1:** Review PICo criteria.

Population	Intervention	Context
Adults (>18 yrs) Acute ischaemic stroke Primary intracerebral haemorrhage Diagnosis or suspected early post-stroke seizures	Seizure identification/monitoring	Receiving inpatient hospital care for acute stroke Less than 2 weeks post stroke onset

**Table 2:** Characteristics of papers reporting on methods used for the identification and observation of early post-stroke seizures.

Variables	Count
**Year of publication (all papers)**	
1996–2001	1
2002–2007	4
2008–2013	5
2014–2019	7
2020–2023	13
**Continent of publication (all papers)**	
Europe	18
North America	9
Asia	2
Africa	1
**Clinical setting (17 research papers)**	
Neurology/neuroscience	3
Critical care/intensive care	3
Stroke specialist	8
Setting not reported	3
**Stroke type (17 research papers)**	
Intracerebral haemorrhage	1
Ischaemic stroke	9
Mixed sample	7
**Sample size (17 research papers)**	
0–25	0
26–50	1
51–100	3
101–200	5
201–300	2
301–500	4
501–1000	0
>1001	2

**Table 3:** Expert opinion papers and case reports detailing methods used for identification and observation of early post-stroke seizures.

First Author, Year	Paper Type	Country	Clinical Setting	Clinical Method to Identify Seizure	Duration of Method	Indications
**Algin, 2022** [24]	Case report	Turkey	Neurology	Clinical observation of focal seizure + periodic EEG	Multiple EEG results. Details on timing and type not reported.	Focal seizure observed + subsequent depression in general conscious level
**Bautista, 2020** [25]	Expert opinion	USA	Critical care	Nurse seizure assessment Bedside EEG recording	Continuous EEG for first 5 min of the seizure and until returned to baseline	On admission EEG as soon as possible after ictal event
**Cyrous, 2012** [26]	Expert opinion	Sweden	Acute stroke	EEG + continuous EEG	Not reported	For ICU stroke patients who are comatose or sedated and paralysed
**De Reuck, 2009** [27]	Expert opinion	Belgium	Stroke	EEG	Not reported	As soon as possible after the ictal event
**Elmali, 2021** [28]	Case report	Turkey	ED	Emergency EEG + continuous video EEG	Emergency EEG details not reported. Continuous video EEG for 48 h.	Episodes of fluctuating confusion suggestive of seizures
**Kraus, 2002** [29]	Expert opinion	USA	Critical care	Continuous EEG	Not reported	For ICU stroke patients who are comatose
**Mader, 2020** [30]	Case report	USA	Not reported	Continuous EEG	48 h, days 2 and 3 post-stroke	Not reported
**Vespa, 2005** [31]	Review and expert opinion	USA	Critical care	Continuous EEG on monitor at bedside, nurse continuous review with routine periodic review by physician	5 days	Lack of clinical seizure activity not an indication to avoid continuous EEG (cEEG)
**Wang, 2021** [32]	Case report	China	Neurology	Video EEG	Not reported	Persistent coma 4 days post-stroke
**Zelano, 2020** [2]	Review and expert opinion	Sweden	Not reported	Continuous EEG + concurrent video recording	≥24 h In patients who are comatose, have periodic discharges, or sedated ≥48 h monitoring advised	As soon as possible after sudden onset of unexplained behavioural changes, or impairment of consciousness (including coma), or clinical paroxysmal events suspected to be seizures

**Table 6:** Types of inpatient clinical methods for the identification and observation of early post-stroke seizures.

First Author, Year	Method Description	Initiation Timing	Duration	Indication
**Continuous EEG**				
**Bautista, 2020** [25]	Observe bedside EEG similar to cardiac monitoring. EEG should be reviewed for frequency, repetition, amplitude, distribution, timing, persistence, morphology, symmetry.	Not reported	Not reported	Not reported
**Belcastro, 2014** [43]	Prolonged > 6 h VEEG. Cap with 21 fixed gel electrodes, International 10–20 system.	Within the first week: promptly in those with clear or suspected seizure, or routinely at any time	>6 h	Routinely in all patients within the first week of admission. Performed promptly if seizure suspected.
**Bentes, 2017** [44]	64-channel VEEG, including an eyes closed wake resting condition and eyes open, hyperventilation, and photic stimulation manoeuvres	First 72 h after stroke	Maximum 60 min	All patients received routine EEG
**Bentes, 2018** [13]	64-channel synchronised VEEG	As early as possible in the first 72 h, daily for the first 7 days	Maximum 60 min	All patients received routine EEG for the first 7 days. Additional VEEG if unexplained neurological worsening.
**Carrera, 2006** [45]	10 electrodes, International 10–20 system with 8 channel sub-set. Displayed continuously at the bedside.	After 24 h of admission	Typically started in the morning and stopped the following day	All patients admitted for longer than 24 h
**Cyrous, 2012** [26]	Not reported	Not reported	Not reported	In the intensive care unit
**Elmali, 2021** [28]	Long-term VEEG	Within first days of admission	48 h	After seizures observed in the emergency department and emergency EEG performed
**Hemphill, 2015** [49]	Not reported	Not reported	Not reported	In ICH patients with depressed mental status that is out of proportion to the degree of brain injury
**Kraus, 2002** [29]	Not reported	Not reported	Not reported	Not reported
**Mader, 2020** [30]	Not reported	At stroke onset	Not reported	Depressed level of consciousness
**Onder, 2017** [40]	Continuous VEEG monitoring 10–20 system	Not reported	1–38 days (mean 7.9)	In a neurological intensive care unit in patients with suspected seizures, unexplained alterations in consciousness or behaviour, or witnessed seizures
**Scoppettulo, 2019** [37]	cEEG 21 scalp electrodes, International 10–20 system	0.5 to 4 days	Not reported	Neurological deterioration: worse neurological deficit increase in NIHSS ≥2 points; fluctuating mental state or drop in GCS ≥ 1 point; new clinical symptoms not attributable to the initial stroke lesion
**Sheikh, 2020** [51]	cEEG, 21 disc electrodes, International 10–20 system	Admission day 1–3	≥6 h	Based on clinical indication
**Tatum, 2022** [50]	Standard configurations apply the International 10–20 system in common bipolar and referential montages for clinical EEG. A minimum of 16 channels for diagnostic long-term VEEG monitoring. Consensus endorsed using more than the 21 electrodes of the International 10–20 system of electrode placement.	Not reported	Duration will vary relative to the indication for performance and number of seizures and events captured	Long-term VEEG monitoring should be used to differentiate between epileptic and non-epileptic events in patients where the diagnosis is in question
**Vespa, 2003** [48] **and 2005** [31]	14 channel EEG. At bedside with monitor for nurse to observe. Physician trained in EEG interpretation review EEG at least three times per day and when nurse identifies suspicious activity. Seizures were detected in one of three ways: on-line identification of seizures by the neuro-ICU nurse or neuro-intensivist, by the total power trend seizure detection method, or by detection during regularly scheduled EEG segment review.	Earliest opportunity after admission to ICU	5 to 7 days	If resource are limited, intracerebral haemorrhage should have priority over ischaemic stroke due to higher risk Lack of clinical seizure activity not an indication to avoid EEG
**Wang, 2021** [32]	VEEG monitoring	Four days post stroke	Not reported	With EEG monitoring. Initiated due to persistent coma after observed seizure and continued until EEG abnormalities had disappeared.
**Yerram, 2019** [39]	Continuous VEEG, 21 electrodes, International 10–20 system	Not reported	Not reported	Based on indication by the physician
**Zelano, 2020** [2]	cEEG monitoring	As soon as possible when non-convulsive seizures suspected	At least 24 h recommended. ≥48 h if comatose, has periodic discharges or sedated	Persistently abnormal mental status following clinically diagnosed seizures or generalised convulsive status epilepticus. Unexplained or fluctuant altered mental status. Clinical paroxysmal events suspected to be seizures. Periodic discharges on routine or emergent EEG.
**Periodic EEG**				
**Algin, 2022** [24]	Portable bedside EEG	In the early period	Not reported	Presence of focal clonic seizures and following day increased drowsiness, meaningless gaze, and reduced speech
**Ba, 2021** [33]	Not reported	Not reported	Not reported	EEG used in case of atypical manifestation, no systematic EEG
**Beghi, 2011** [42]	Not reported	At hospitalisation	Within first 7 days	When indicated by the caring physician, according to local practice
**De Reuck, 2009** [27]	Not reported	Not reported	Not reported	EEG performed as soon as possible after ictal event
**Elmali, 2021** [28]	Emergency EEG	Not reported	Not reported	Episodes of fluctuating confusion suggestive of seizures
**Green, 2021** [23]	Not reported	Not reported	Not reported	EEG for change in mental status or depressed level of consciousness out of proportion to the stroke
**Kim, 2016** [36]	Standard EEG	Within 7 days of stroke onset	20–30 min	Performed within 24–72 h of PSSi onset
**Lasek-Bal, 2023** [46]	Standard protocol including hyperventilation and photo stimulation. Conducted at rest and supine. Galileo EEG–EP device with 21 electrodes, International 10–20 system.	Within first 72 h of admission	20 min	Routinely performed on all eligible patients
**Mecarelli, 2011** [47]	Micromed digital device, 14 disc electrodes, International 10–20 system	Within 24 h of admission	30 min minimum	If status epilepticus detected, EEG continued to monitor pharmacological treatment. If first EEG showed abnormal epileptiform activity, series of EEGs were performed over the following days. EEG available weekdays only.
**Tako, 2022** [38]	Not reported	Not reported	Not reported	Performed according to the indication of the attending physician
**Clinician Observation**				
**Algin, 2022** [24]	Seizure suspected after increased drowsiness, meaningless gaze, and reduced speech. No seizure observed.	Not reported	Not reported	Not reported
**Bautista, 2020** [25]	Monitor airway, level of consciousness, eye deviation, gaze, pupil size, urinary incontinence, body movements, and motor function. Responsiveness, awareness, motor function, and language should be assessed in ictal and postictal phase. Record onset and duration of seizure.	Not reported	During first 5 min observe continuously. Observe in ictal and post ictal phases until patient back at their baseline	Not reported
**Beghi, 2011** [42]	Direct observation by medical staff at time of admission or from reliable witness history (e.g., ambulance personnel)	At hospitalisation	Within the first 7 days	Not reported
**Daniele, 1996** [34]	Diagnosis of seizure based on observation and description by experienced staff from our department.	Not reported	Not reported	Not applicable
**Green, 2021** [23]	Nurses should have a standardised approach for recognition of seizures. Assessment and documentation of the seizure	Not reported	Not reported	Not reported
**Jung, 2012** [35]	Patient or witness reporting, or both. Seizure type and time of occurrence.	At stroke onset	Within first 24 h	Seizures in basilar artery occlusion were only assumed if further signs like unequivocal clonic movements, tongue bite, or incontinence were observed
**Kim, 2016** [36]	Seizure was distinguished as being partial or generalized, according to the 2010 ILAE criteria	Within first 7 days	Within first 7 days	Simple loss of consciousness or short-lasting episodes of mental confusion were not considered for epileptic seizure diagnosis
**Sarfo, 2021** [41]	Seizures diagnosed clinically and recorded in medical notes or witness reporting	At hospitalisation	Within first 7 days of stroke onset	Classified as acute symptomatic seizures according to ILAE criteria, and focal or generalised
**Tako, 2022** [38]	Acute symptomatic seizures diagnosed by clinically observed ictal stigmas	Not reported	Within first 7 days of stroke onset	Classified as acute symptomatic seizures according to ILAE criteria, further seizure classification dependent on EEG findings
**VEEG**				
**Belcastro, 2014** [43]	Video recording alongside continuous EEG	Within the first week: promptly in those with clear or suspected seizure, or routinely at any time	>6 h	Routinely in all patients within the first week of admission
**Bentes, 2017** [44]	Video recording alongside continuous EEG	First 72 h after stroke	Maximum 60 min	All patients received routine EEG
**Bentes, 2018** [13]	Video recording alongside continuous EEG	As early as possible in the first 72 h, daily for the first 7 days	Maximum 60 min	All patients received video EEG for the first 7 days. Additional video EEG if unexplained neurological worsening.
**Elmali, 2021** [28]	Long-term video EEG	Within first days of admission	48 h	After seizures observed in the emergency department and emergency EEG performed
**Mader, 2020** [30]	Video recording alongside EEG	At stroke onset, duration not reported	Not reported	Continuous with EEG
**Onder, 2017** [40]	Video recording alongside cEEG	Not reported	1–38 days (mean 7.9)	With cEEG
**Tatum, 2022** [50]	One camera is usual practice. Use more than 21 electrodes of the International 10–20 system of electrode placement. Standard digital audio–video data is acquired, provided by standard industry codecs. Specification of time synchronisation between video and EEG has been standardised in the DICOM format and MED format. 24 h VEEG requires up to 30 GB memory.	Not reported	Duration will vary relative to the indication for performance and number of seizures and events captured	Long-term VEEG monitoring should be used to differentiate between epileptic and non-epileptic events in patients where the diagnosis is in question
**Wang, 2021** [32]	VEEG monitoring	Four days post stroke	Not reported	With EEG monitoring. Initiated due to persistent coma after observed seizure.
**Yerram, 2019** [39]	Video recording alongside cEEG	Not reported	Not reported	With cEEG
**Zelano, 2020** [2]	Concurrent video recording is strongly recommended as supplementary to neurologic examination to evaluate clinical behaviour and to assess whether electrographic seizures are associated with clinical changes	In the early phase	Not reported	With cEEG
**Family Witness**				
**Beghi, 2011** [42]	As well as other detection methods, diagnosis of seizure was based on history according to reliable witness description	At hospitalisation	Within the first 7 days	Not appliable
**Daniele, 1996** [34]	The diagnosis of epileptic seizure was performed by observation, including description by relatives of the patients who witnessed it	Not reported	Not reported	Not applicable
**Jung, 2012** [35]	Information obtained from the patient or persons who observed the seizure, or both	From stroke onset	Until 3 month post-stroke follow-up	Not applicable
**Mader, 2020** [30]	Relative noted 30 s episode of bilateral leg jerking 30 min after drop in level of consciousness	30 min after change in neurology indicating acute stroke	Not applicable	Not applicable
**Sarfo, 2020** [41]	As well as clinical observation, often a family member witnessed symptoms at time of presentation	On hospitalisation	Within the first 7 days	Not applicable

**Table 7:** Indications for performing EEG reported in the literature.

Indications for EEG (References) *	Indication Frequency Based on Number of Papers and Type of Publication
Routine at prescribed timepoints post-stroke [13,30,31,43,44,45,46,47,48]	9 (7 research, 1 case report, 1 opinion paper)
Depression in conscious level or coma [2,23,24,26,29,32,40]	7 (3 opinion papers, 1 case report, 1 guideline paper, 1 research)
After direct observation of seizure [24,25,27,33,36,42]	6 (3 research, 2 opinion papers, 1 case report)
Fluctuating confusion or unexplained behavioural changes [2,23,28,49]	4 (1 case report, 1 opinion paper, 2 guideline papers)
Indicated by medical staff [38,39,42]	3 (3 research papers)
Atypical seizure manifestation [33]	1 (1 research paper)
Paroxysmal events suspected to be seizures [2]	1 (1 opinion paper)
Neurological deterioration [37]	1 (1 research paper)
Condition specific [50]	1 (1 guideline paper)
* Studies excluded as EEG not referred to in paper [34,35,41]	3 (3 research papers)

## Data Availability

No new data were created or analyzed in this study. Data sharing is not applicable.

## Supplementary Materials

The following supporting information can be downloaded at: https://www.mdpi.com/article/10.3390/neurolint17100159/s1. Supplementary Material 1: search strategies for MEDLINE (Ovid), CINAHL (EBSCOhost), EMBASE (Ovid), and the Cochrane Library; Table S1: Preferred Reporting Items for Systematic reviews and Meta-Analyses extension for Scoping Reviews (PRISMA-ScR) checklist.

## Abbreviations

The following abbreviations are used in this manuscript:

EPSS	Early post-stroke seizure
EEG	Electroencephalogram
cEEG	Continuous electroencephalogram
VEEG	Video-electroencephalogram
