# Supplementary material for: Clinical Methods Supporting Initial Recognition of Early Post-Stroke Seizures: A Systematic Scoping Review
Source: Neurol Int. 2025 Oct 3;17(10):159. doi: 10.3390/neurolint17100159 (PMC12567525; doi:10.3390/neurolint17100159)
Supplement: Supplementary file 1 [file neurolint-17-00159-s001.zip › neurolint-3797082-supplementary.pdf]

Figure S1 Search Strategies

Database: Ovid MEDLINE(R) and Epub Ahead of Print, In-Process, In-Data-Review & Other Non-Indexed Citations and Daily 1946 to September 30, 2021

Date searched: 01/10/2021

1 Epilepsy/

2 Seizures/

3 Status Epilepticus/

4 1 or 2 or 3

5 exp Stroke/

6 exp Cerebral Hemorrhage/

7 5 or 6

8 4 and 7

9 (Post-stroke seizure* or Poststroke seizure* or Post-stroke epilep* or Poststroke epilep* or postisch?emic stroke epilep* or post-isch?emic stroke epilep* or postisch?emic stroke seizure* or post-isch?emic stroke seizure* or postisch?emic seizure* or post-isch?emic seizure* or postisch?emic epilep* or post-isch?emic epilep* or posth?emorrhagic stroke epilep* or post-h?emorrhagic stroke epilep* or posth?emorrhagic stroke seizure* or post-h?emorrhagic stroke seizure* or acute symptomatic seizure*).ti,ab,kw.

10 ((seizure* or convuls* or epilep*) and (stroke* or poststroke or post-stroke or cerebrovascular accident* or cva or intracerebral h?emorrhage* or cerebral h?emorrhage* or brain h?emorrhage* or cerebral infarction* or brain infarction*)).ti.

11 ((seizure* or epilep* or convuls*) adj6 (stroke* or poststroke or post-stroke or cerebrovascular accident* or cva or intracerebral h?emorrhage* or cerebral h?emorrhage* or brain h?emorrhage* or cerebral infarction* or brain infarction*) adj6 (related or associated or follow* or after or onset or caus* or during or occur* or within)).ab.

12 8 or 9 or 10 or 11

13 limit 12 to english language

14 exp animals/ not humans.sh.

15 13 not 14

16 (exp child/ or exp infant/ or exp adolescent/) not exp Adult/

17 15 not 16

Database: Embase (Ovid) 1974 to 2021 September 30

Date searched: 01/10/2021

1 epilepsy/

2 seizure/

3 epileptic state/

4 1 or 2 or 3

5 cerebrovascular accident/

6 brain infarction/

7 brain hemorrhage/

8 5 or 6 or 7

9 4 and 8

10 (Post-stroke seizure* or Poststroke seizure* or Post-stroke epilep* or Poststroke epilep* or postisch?emic stroke epilep* or post-isch?emic stroke epilep* or postisch?emic stroke seizure* or post-isch?emic stroke seizure* or postisch?emic seizure* or post-isch?emic seizure* or postisch?emic epilep* or post-isch?emic epilep* or posth?emorrhagic stroke epilep* or post-h?emorrhagic stroke epilep* or posth?emorrhagic stroke seizure* or post-h?emorrhagic stroke seizure* or acute symptomatic seizure*).ti,ab,kw.

11 ((seizure* or convuls* or epilep*) and (stroke* or poststroke or post-stroke or cerebrovascular accident* or cva or intracerebral h?emorrhage* or cerebral h?emorrhage* or brain h?emorrhage* or cerebral infarction* or brain infarction*)).ti.

12 ((seizure* or epilep* or convuls*) adj6 (stroke* or poststroke or post-stroke or cerebrovascular accident* or cva or intracerebral h?emorrhage* or cerebral h?emorrhage* or brain h?emorrhage* or cerebral infarction* or brain infarction*) adj6 (related or associated or follow* or after or onset or caus* or during or occur* or within)).ab.

13 9 or 10 or 11 or 12

14 limit 13 to english language

15 (rat or rats or mouse or mice or swine or porcine or murine or sheep or lambs or pigs or piglets or rabbit or rabbits or cat or cats or dog or dogs or cattle or bovine or monkey or monkeys or trout or marmoset$1).ti. and animal experiment/

16 Animal experiment/ not (human experiment/ or human/)

17 15 or 16

18 14 not 17

19 (exp child/ or exp adolescence/ or exp adolescent/) not exp adult/

20 18 not 19

Database: CINAHL Complete (via EBSCOhost)

Date searched: 01/10/2021

S1 (MH "Epilepsy")

S2 (MH "Seizures")

S3 (MH "Status Epilepticus")

S4 S1 OR S2 OR S3

S5 (MH "Stroke+")

S6 (MH "Cerebral Hemorrhage+")

S7 S5 OR S6

S8 S4 AND S7

S9 "Post-stroke seizure*" or "Poststroke seizure*" or "Post-stroke epilep*" or "Poststroke epilep*" or "postisch#emic stroke epilep*" or "post-isch#emic stroke epilep*" or "postisch#emic stroke seizure*" or "post-isch#emic stroke seizure*" or "postisch#emic seizure*" or "post-isch#emic seizure*" or "postisch#emic epilep*" or "post-isch#emic epilep*" or "posth#emorrhagic stroke epilep*" or "post-h#emorrhagic stroke epilep*" or "posth#emorrhagic stroke seizure*" or "post-h#emorrhagic stroke seizure*" or "acute symptomatic seizure*"

S10 TI ((seizure* or convuls* or epilep*) and (stroke* or poststroke or "post-stroke" or "cerebrovascular accident*" or cva or "intracerebral h#emorrhage*" or "cerebral h#emorrhage*" or "brain h#emorrhage*" or "cerebral infarction*" or "brain infarction*"))

S11 AB ((seizure* or epilep* or convuls*) N6 (stroke* or poststroke or "post-stroke" or "cerebrovascular accident*" or cva or "intracerebral h#emorrhage*" or "cerebral h#emorrhage*" or "brain h#emorrhage*" or "cerebral infarction*" or "brain infarction*") N6 (related or associated or follow* or after or onset or caus* or during or occur* or within))

S12 S8 OR S9 OR S10 OR S11

S13 MH animals+

S14 MH (animal studies)

S15 TI (animal model*)

S16 S13 OR S14 OR S15

S17 MH (human)

S18 S16 NOT S17

S19 S12 NOT S18

S20 (MH "Child+")

S21 (MH "Infant+")

S22 (MH "Adolescence+")

S23 S20 OR S21 OR S22

S24	(MH "Adult+")
S25	S23 NOT S24
S26	S19 NOT S25
S27	S19 NOT S25 limited to English Language

Database: Cochrane Library via Wiley (all databases)

Date searched: 01/10/2021

#1 MeSH descriptor: [Epilepsy] explode all trees

#2 MeSH descriptor: [Seizures] this term only

#3 MeSH descriptor: [Status Epilepticus] this term only

#4 #1 OR #2 OR #3

#5 MeSH descriptor: [Stroke] explode all trees

#6 MeSH descriptor: [Cerebral Hemorrhage] explode all trees

#7 #5 OR #6

#8 #4 AND #7

#9 (("post stroke" or poststroke or postischemic or postischaemic or "post-ischemic" or "post-ischaemic" or "postischemic Stroke" or "post-ischemic Stroke" or "postischaemic stroke" or "post-ischaemic stroke" or "posthemorrhagic stroke" or "post-hemorrhagic stroke" or "posthaemorrhagic stroke" or "post-haemorrhagic stroke") NEXT (epilep* or seizure*)):ti,ab,kw

#10 ("acute symptomatic seizure" or "acute symptomatic seizures"):ti,ab,kw

#11 ((seizure* or convuls* or epilep*) and (stroke* or poststroke or "post-stroke" or (cerebrovascular NEXT accident*) or cva or (intracerebral NEXT h?emorrhage*) or (cerebral NEXT h?emorrhage*) or (brain NEXT h?emorrhage*) or (cerebral NEXT infarction*) or (brain NEXT infarction*))):ti

#12 (((seizure* or epilep* or convuls*) NEAR/6 (stroke* or poststroke or "post-stroke" or (cerebrovascular NEXT accident*) or cva or (intracerebral NEXT h?emorrhage*) or (cerebral NEXT h?emorrhage*) or (brain NEXT h?emorrhage*) or (cerebral NEXT infarction*) or (brain NEXT infarction*)) NEAR/6 (related or associated or follow* or after or onset or caus* or during or occur* or within))):ab

#13 #8 OR #9 OR #10 OR #11 OR #12

#14 MeSH descriptor: [Animals] explode all trees

#15 MeSH descriptor: [Humans] explode all trees

#16 #14 NOT #15

#17 #13 NOT #16

#18 MeSH descriptor: [Child] explode all trees

#19 MeSH descriptor: [Infant] explode all trees

#20 MeSH descriptor: [Adolescent] explode all trees

#21 #18 OR #19 OR #20

#22 MeSH descriptor: [Adult] explode all trees

#23 #21 NOT #22

#24 #17 NOT #23

Table S1 Preferred Reporting Items for Systematic reviews and Meta-Analyses extension for Scoping Reviews (PRISMA-ScR) Checklist

SECTION	ITEM	PRISMA-ScR CHECKLIST ITEM	REPORTED ON PAGE #
**TITLE**			
Title	1	Identify the report as a scoping review.	1
**ABSTRACT**			
Structured summary	2	Provide a structured summary that includes (as applicable): background, objectives, eligibility criteria, sources of evidence, charting methods, results, and conclusions that relate to the review questions and objectives.	1
**INTRODUCTION**			
Rationale	3	Describe the rationale for the review in the context of what is already known. Explain why the review questions/objectives lend themselves to a scoping review approach.	2
Objectives	4	Provide an explicit statement of the questions and objectives being addressed with reference to their key elements (e.g., population or participants, concepts, and context) or other relevant key elements used to conceptualize the review questions and/or objectives.	2-3
**METHODS**			
Protocol and registration	5	Indicate whether a review protocol exists; state if and where it can be accessed (e.g., a Web address); and if available, provide registration information, including the registration number.	2
Eligibility criteria	6	Specify characteristics of the sources of evidence used as eligibility criteria (e.g., years considered, language, and publication status), and provide a rationale.	3
Information sources*	7	Describe all information sources in the search (e.g., databases with dates of coverage and contact with authors to identify additional sources), as well as the date the most recent search was executed.	3
Search	8	Present the full electronic search strategy for at least 1 database, including any limits used, such that it could be repeated.	3
Selection of sources of evidence†	9	State the process for selecting sources of evidence (i.e., screening and eligibility) included in the scoping review.	3-4
Data charting process‡	10	Describe the methods of charting data from the included sources of evidence (e.g., calibrated forms or forms that have been tested by the team before their use, and whether data charting was done independently or in duplicate) and any processes for obtaining and confirming data from investigators.	4
Data items	11	List and define all variables for which data were sought and any assumptions and simplifications made.	4
Critical appraisal of individual sources of evidence§	12	If done, provide a rationale for conducting a critical appraisal of included sources of evidence; describe the methods used and how this information was used in any data synthesis (if appropriate).	N/a
Synthesis of results	13	Describe the methods of handling and summarizing the data that were charted.	4
**RESULTS**			

SECTION	ITEM	PRISMA-ScR CHECKLIST ITEM	REPORTED ON PAGE #
Selection of sources of evidence	14	Give numbers of sources of evidence screened, assessed for eligibility, and included in the review, with reasons for exclusions at each stage, ideally using a flow diagram.	4
Characteristics of sources of evidence	15	For each source of evidence, present characteristics for which data were charted and provide the citations.	5
Critical appraisal within sources of evidence	16	If done, present data on critical appraisal of included sources of evidence (see item 12).	N/a
Results of individual sources of evidence	17	For each included source of evidence, present the relevant data that were charted that relate to the review questions and objectives.	6-14
Synthesis of results	18	Summarize and/or present the charting results as they relate to the review questions and objectives.	14-15
**DISCUSSION**			
Summary of evidence	19	Summarize the main results (including an overview of concepts, themes, and types of evidence available), link to the review questions and objectives, and consider the relevance to key groups.	15
Limitations	20	Discuss the limitations of the scoping review process.	15
Conclusions	21	Provide a general interpretation of the results with respect to the review questions and objectives, as well as potential implications and/or next steps.	16
**FUNDING**			
Funding	22	Describe sources of funding for the included sources of evidence, as well as sources of funding for the scoping review. Describe the role of the funders of the scoping review.	16

JBI = Joanna Briggs Institute; PRISMA-ScR = Preferred Reporting Items for Systematic reviews and Meta-Analyses extension for Scoping Reviews.

From: Tricco AC, Lillie E, Zarin W, O'Brien KK, Colquhoun H, Levac D, et al. PRISMA Extension for Scoping Reviews (PRISMAScR): Checklist and Explanation. Ann Intern Med. 2018;169:467–473. doi: 10.7326/M18-0850.
